# Genome-wide characterization of BPC transcription factors in pear and functional validation of *PbBPC5* in drought tolerance regulation

**DOI:** 10.3389/fpls.2025.1752990

**Published:** 2026-01-21

**Authors:** Xin Jia, Xing Han, Yuan Cheng, Xiaoli Ren, Guiyan Fan, Xiaocong Jiao, Yueyue Cai, Lu Li, Chenwei Zhang, Hongguang Pang

**Affiliations:** 1 Modern Agricultural Science and Technology Laboratory, Department of Agriculture and Food Science, Shijiazhuang University, Shijiazhuang, China; 2 Hebei International Joint Research Center for Green Agricultural Biological Agents, Department of Agriculture and Food Science, Shijiazhuang University, Shijiazhuang, China

**Keywords:** BPC transcription factor, drought stress, genome-wide analysis, pear, ROS

## Abstract

**Introduction:**

The BASIC PENTACYSTEINE (BPC) family comprises plant-specific transcription factors that regulate diverse developmental programs and stress responses. Pear (*Pyrus bretschneideri*), an economically significant fruit crop, often experiences marked declines in fruit yield and quality under drought stress. Although *BPC* genes have been identified in several plant species, a comprehensive characterization of this family in pear is lacking.

**Methods:**

In this study, we systematically characterized *PbBPC* genes in the pear genome using various bioinformatic approaches. We examined their expression profiles across diverse tissues and under dehydration conditions and further validated the role of *PbBPC5* in drought tolerance using virus-induced gene silencing (VIGS).

**Results:**

This study identified seven *PbBPC* genes in the pear genome, which were subsequently classified into three distinct groups through phylogenetic analysis. Comprehensive bioinformatics analyses were performed, examining their phylogenetic relationships, gene structures, conserved motifs, protein domains, chromosomal locations, and gene duplication events. Promoter analyses showed that all *PbBPC* genes contained various *cis*-acting elements associated with growth and development, stress response, and phytohormone signaling. Quantitative real-time PCR (qRT-PCR) showed that most *PbBPC* transcripts were upregulated by dehydration, with *PbBPC5* exhibiting the strongest upregulation. Furthermore, subcellular localization experiments indicated that PbBPC5 was localized to the nucleus. Silencing *PbBPC5* reduced drought tolerance, as indicated by more severe wilting under water deficit, lower relative water content, higher electrolyte leakage, and elevated malondialdehyde levels. *PbBPC5* silencing also weakened antioxidant defenses during drought by reducing antioxidant enzyme activities. These results suggest that *PbBPC5* functions on drought tolerance regulation in pear mainly by influencing reactive oxygen species scavenging.

**Discussion:**

This study provides a genome-wide characterization of the *PbBPC* family and reveals *PbBPC5* as a key regulator of the drought response, offering a foundation for improving pear drought tolerance through genetic approaches.

## 1 Introduction

Pear (*Pyrus bretschneideri*) is an economically, nutritionally, and medicinally important fruit crop. However, climate change and groundwater scarcity increasingly expose pear trees to drought stress, which negatively affects fruit yield and quality (Lin et al., 2025). Identifying drought-responsive genes is essential for breeding drought-resistant cultivars through genetic improvement and for supporting the sustainable development of the pear industry.

The BASIC PENTACYSTEINE (BPC)/BARLEY B RECOMBINANT (BBR) gene family comprises plant-specific transcription factors (TFs) that regulate diverse developmental processes (Ma et al., 2022; Sahu et al., 2023). BPC TFs, also known as GAGA-binding proteins, recognize and bind to GAGA repeat sequences or C-box elements (RGARAGRRAA) in gene promoter regions (Meister et al., 2004; Kooiker et al., 2005; Hecker et al., 2015). In Arabidopsis, approximately 7% of the 3-kb upstream regions of annotated genes contain at least one (GA)6 motif, and nearly 80% contain a C-box (Hecker et al., 2015). The widespread distribution of these motifs suggests that BPC TFs regulate the transcription of large numbers of genes. To date, seven BPC members have been identified in Arabidopsis. All of these proteins share a conserved C-terminal region containing five cysteine residues that recognize GA-rich boxes or C-boxes in target gene promoters (Monfared et al., 2011; Theune et al., 2019). The Arabidopsis BPC proteins are classified into three groups based on sequence similarity and N-terminal structural features: class I (AtBPC1–3), class II (AtBPC4–6), and class III (AtBPC7) (Monfared et al., 2011; Petrella et al., 2020).

BPC TFs regulate a broad range of developmental processes, including seed and ovule development, bud dormancy, endosperm growth, vegetative-to-reproductive transitions, sex determination, stomatal and leaf morphogenesis, root development, inflorescence formation, fertility, and fruit development (Hecker et al., 2015; Ezquer et al., 2016; Charlesworth, 2021; Iwasaki et al., 2021; Lloret et al., 2021; Sahu et al., 2023). The quadruple *atbpc1 atbpc2 atbpc4 atbpc6* mutant exhibits extensive defects in both vegetative and reproductive growth (Monfared et al., 2011). In rice, knockout of *OsBPC1* promotes seedling growth and increases grain length, highlighting its negative role in regulating yield (Gong et al., 2018). In Arabidopsis, BPCs modulate lateral root development by repressing *ABSCISIC ACID INSENSITIVE4* (*ABI4*) expression, linking their function to the auxin pathway (Mu et al., 2017a). In cucumber, CsBPC proteins bind to the *ABI3* promoter to inhibit its expression, thereby influencing seed germination (Mu et al., 2017b). Knockout of *CsBPC2* further impairs root growth by disrupting gibberellin biosynthesis (Feng et al., 2023). In addition, *AtBPC3* contributes to circadian rhythm regulation and leaf edge formation in *Arabidopsis thaliana* (Lee et al., 2022). In apple, MdBPC2 interacts with LIKE HETEROCHROMATIN PROTEIN 1 (LHP1) to repress the expression of two auxin synthesis genes (*MdYUC2a* and *MdYUC6b*), thereby modulating plant height, leaf morphology, and root growth (Zhao et al., 2024). In *Marchantia*, BPC TFs are also critical regulators of sex determination (Iwasaki et al., 2021). Many of these developmental roles are mediated through phytohormone signaling, including ethylene, abscisic acid (ABA), and cytokinin (Monfared et al., 2011; Simonini and Kater, 2014; Mu et al., 2017a; Shanks et al., 2018).

The functions of BPC TFs in stress responses are less understood compared with their developmental roles. Previous studies have reported that BPC gene expression can be induced by diverse stresses in flowering Chinese cabbage, cucumber, and oilseed rape (Li et al., 2019; Zhang et al., 2023; Wang et al., 2024). In Arabidopsis, *BPC1* and *BPC2* enhance salt tolerance by reducing β-1,4-galactan accumulation in the cell wall (Yan et al., 2021). Conversely, *AtBPC2* negatively regulates tolerance to mannitol during the seedling stage by repressing a late embryogenesis abundant gene (*LEA4-5*) (Li et al., 2021). In tobacco, overexpression of *CsBPC2* inhibits seed germination under NaCl and PEG treatment (Li et al., 2019). More recently, *CsBPC2* was shown to promote salt tolerance by enhancing osmoprotectant biosynthesis, reactive oxygen species (ROS) scavenging, ion homeostasis, and ABA signaling (Li et al., 2023). Similarly, *BcBPC9* has been identified as a positive regulator of cadmium stress resistance in flowering Chinese cabbage (Zhang et al., 2023). Collectively, these studies suggest that BPC TFs are involved in the responses of plants to multiple types of stress, though additional research is needed to clarify their mechanisms.

Members of the BPC gene family have been identified in several species, such as coconut palm, grape, and oilseed rape, broadening our understanding of this TF family (Lao et al., 2024; Hu et al., 2025; Zhang et al., 2025). However, little is known about BPCs in pear. In this study, we identified seven *PbBPC* genes from the Chinese white pear genome and analyzed their sequence features, phylogenetic relationships, protein motifs, gene structures, promoter *cis*-elements, chromosomal distributions, and collinearity. We further examined their expression patterns in different tissues and under dehydration treatment. *PbBPC5* silencing via virus-induced gene silencing (VIGS) reduced drought tolerance in pear. Overall, we provide the first comprehensive characterization of the BPC family in pear, uncover a role for *PbBPC5* in the drought response, and offer candidate genetic resources for breeding drought-resistant cultivars through genetic engineering.

## 2 Materials and methods

### 2.1 Identification of *BPC* gene family members in pear

To identify BPC gene family members in pear (*Pyrus bretschneideri* Rehd.), we first retrieved genome and proteome sequences of pear, apple (*Malus domestica*), and peach (*Prunus persica*) from the Rosaceae Genome Database (https://www.rosaceae.org/). BPC protein sequences from *Arabidopsis thaliana* were downloaded from the TAIR database (https://www.arabidopsis.org/) and used as queries to identify all putative BPC homologs in the genomes of pear, apple, and peach using BLASTp with an e-value of ≤ 1e^−5^. Subsequently, the hidden Markov model (HMM) of the GAGA_bind domain (PF06217) was downloaded from the Pfam database (http://pfam.xfam.org/) and employed to identify *BPC* genes using HMMER 3.0 software with default parameters (http://hmmer.org/). Finally, all putative BPC genes were verified using SMART databases. Protein lengths and genomic information for *PbBPC* genes were obtained from the NCBI database (https://www.ncbi.nlm.nih.gov/). Fundamental physicochemical properties, including isoelectric point (pI), molecular weight, and instability index, were calculated using the ExPASy online tool (https://www.expasy.org/). Subcellular localization of the predicted proteins was assessed using WoLF PSORT (https://wolfpsort.hgc.jp/).

### 2.2 Analysis of phylogenetic relationships, gene structures, and conserved motifs

Multiple sequence alignments of BPC proteins were carried out using MAFFT v7.526 with default settings (Katoh and Standley, 2013). A maximum-likelihood (ML) phylogenetic tree was then constructed using the resulting alignment in IQ-TREE2 v2.3.4 with the parameters “-m MFP -B 1000 –bnni”. The final phylogenetic tree was visualized using the Interactive Tree of Life (iTOL) web server (https://itol.embl.de). The exon–intron structures of *PbBPC* genes were illustrated using the Gene Structure Display Server (https://gsds.gao-lab.org/) based on an annotation file of all *PbBPC* genes in pear. Conserved motifs within *PbBPC* proteins were identified using MEME v5.0.3 with default settings (Bailey et al., 2015).

### 2.3 Chromosomal locations and collinearity analysis

To measure the chromosomal distribution of *PbBPC* members, genome annotation files were obtained from the NCBI database, and their positions were mapped using TBtools (Chen et al., 2020). Collinearity analysis was carried out using the MCScanX toolkit with default parameters to analyze gene duplication events (Wang et al., 2012). The rates of synonymous (Ks) and nonsynonymous (Ka) substitutions were estimated using DnaSP v5.10.01 (Librado and Rozas, 2009).

### 2.4 *Cis*-acting elements in the promoters of *PbBPC* genes

Promoter regions of *PbBPC* genes were analyzed for *cis*-acting regulatory elements utilizing the PlantCARE database (https://bioinformatics.psb.ugent.be/webtools/plantcare/html/). For each gene, a 2,000 base pair (bp) sequence upstream of the initiation codon was extracted and submitted to the PlantCARE database for *cis*-acting element prediction. The distribution of these *cis*-acting elements in the promoter regions was subsequently visualized using TBtools.

### 2.5 Expression patterns of *PbBPC* genes in various tissues of pear

The expression profiles of *PbBPC* genes across various pear tissues were analyzed based on the transcriptome data retrieved from the NCBI database (accession number PRJNA498777). The tissues included leaf, stem, bud, petal, sepal, ovary, and fruit. Bud samples were collected during the flower bud differentiation stage; leaf, stem, petal, sepal, and ovary samples were collected at full flowering; and fruit samples were obtained at commercial maturity (Li et al., 2019). Gene expression levels were quantified using featureCounts v2.0.6 and normalized as fragments per kilobase per million (FPKM) values (Liao et al., 2014).

### 2.6 Expression patterns of *PbBPC* genes under dehydration treatment

*P. betulaefolia* seedlings were cultivated in a growth chamber maintained at 23°C, with a 14 h light/10 h dark photoperiod and a light intensity of 100 μmol m^−2^s^−1^. Following a cultivation period of three months, seedlings of *P. betulaefolia* with uniform size were subjected to dehydration by placing them on dry filter paper at a temperature of 26°C. Leaves were collected at intervals of 0, 1, 3, 6, and 12 h, with three independent biological replicates per time point. Total RNA was isolated from the leaves using the Wolact Plant RNA Isolation Kit (Wolact, Hong Kong, China), and first-strand cDNA synthesis was performed using the Thermo RevertAid First Strand cDNA Synthesis Kit (Thermo Fisher Scientific, USA). qRT-PCR was performed using a Roche LightCycler 96 System (Roche Diagnostics, Basel, Switzerland) via the methodology described by Huo et al. (2020). Each experiment was conducted with three biological replicates, and each biological replicate was analyzed in triplicate. Tubulin was used as the internal reference gene. The 2^-ΔΔCt^ method was used to calculate the relative expression levels of *PbBPC* genes. Primers for the target genes and the internal reference gene are shown in **Supplementary Table S1**.

### 2.7 Subcellular localization of PbBPC5

Mature leaves of *P. bretschneideri* were collected for cloning *PbBPC5*. The open reading frame (ORF) of *PbBPC5* was amplified and inserted into the pRI101-GFP vector to construct a subcellular localization vector. The resulting recombinant plasmid (pRI101-*PbBPC5*) was inserted into *Agrobacterium* GV3101 using electroporation. Healthy tobacco leaves were co-infiltrated with GV3101 carrying the recombinant plasmid and a membrane marker (CBL1n::OFP). Nuclear DNA was stained with 4′,6-diamidino-2-phenylindole (DAPI). Subcellular localization of the *PbBPC5* protein was examined using a laser scanning confocal microscope (FV1200, Olympus, Japan).

### 2.8 Plant transformation and drought treatment

To construct a virus-induced gene silencing (VIGS) vector, a 300-bp fragment of the *PbBPC5* ORF was cloned into the tobacco rattle virus (TRV)-based vector pTRV2. The recombinant pTRV2-*PbBPC5* vector and the empty pTRV1 vector were introduced separately into GV3101 by electroporation. Co-infiltration of GV3101 carrying pTRV2-*PbBPC5* and pTRV1 was performed on 45-day-old pear seedlings following a previously described protocol (Han et al., 2023). Control plants were generated by co-infiltrating GV3101 containing empty pTRV2 and pTRV1 vectors. Silencing efficiency was confirmed by quantifying *PbBPC5* transcript levels using qRT-PCR. Seedlings of similar size from both the silenced group (pTRV2-*PbBPC5* plants) and the control group were subjected to 20 days of water deprivation following adequate irrigation. Leaves were sampled and stored at −80 °C for physiological analyses.

### 2.9 Determination of stress-related physiological parameters

Relative electrolyte leakage was assessed in accordance with the protocol outlined by Lutts et al. (1996), while relative water content (RWC) was determined using the methodology described by Gaxiola et al. (2001). Quantification of malondialdehyde (MDA), hydrogen peroxide (H_2_O_2_), and superoxide radical (O_2_^–^), along with the enzymatic activities of superoxide dismutase (SOD), peroxidase (POD), and catalase (CAT), was conducted utilizing commercial assay kits provided by Suzhou Comin Biotechnology Co., Ltd., Suzhou, China. For the histochemical detection of ROS, leaves from both silenced and control plants were harvested at the end of the drought treatment and subjected to staining with nitro blue tetrazolium (NBT) and 3,3-diaminobenzidine (DAB), following established protocols (Fryer et al., 2002).

### 2.10 Statistical analysis

Statistical analyses were performed utilizing SPSS v27.0. Variations among means were assessed through a one-way analysis of variance (ANOVA), and significant differences were identified using Tukey’s multiple range test, with a significance threshold set at *P* < 0.05.

## 3 Results

### 3.1 Genome-wide identification of *BPC* genes in pear

Using the seven Arabidopsis BPC proteins as queries in BLASTp searches against the pear genome, we identified seven *PbBPC* family members. These were verified based on the presence of the conserved BPC domain and designated as *PbBPC1*, *PbBPC2*, *PbBPC3*, *PbBPC4*, *PbBPC5*, *PbBPC6.1*, and *PbBPC6.2*, according to their evolutionary relationships with homologs from other species. As summarized in **Table 1**, the *PbBPC* proteins ranged in size from 279 amino acids (*PbBPC1* and *PbBPC2*) to 340 amino acids (*PbBPC6.1* and *PbBPC6.2*). Their predicted molecular weights spanned from 30,802.48 Da (*PbBPC2*) to 38,137.06 Da (*PbBPC5*). All members exhibited isoelectric points (pI) greater than 9, indicating enrichment in alkaline amino acids. Instability indices ranged from 46.22 (*PbBPC1*) to 65.70 (*PbBPC5*), suggesting that all *PbBPC* proteins are relatively unstable (values exceeding 40 were considered unstable) (Guruprasad et al., 1990). Additionally, all proteins had negative GRAVY values, reflecting their hydrophilic nature. Subcellular localization predictions placed all PbBPC proteins in the nucleus, consistent with their roles as transcription factors (**Table 1**).

**Table 1:** The basic physicochemical properties of *BPC* gene family members in *Pyrus bretschneideri*.

Gene name	Gene ID number	Number of amino acids (aa)	Molecular weight (Da)	Isoelectric point (pI)	Instability index	GRAVY	Subcellular localization
PbBPC1	rna52599	279	30834.53	9.7	46.22	-0.585	nucleus
PbBPC2	rna5540	279	30802.48	9.7	48.1	-0.589	nucleus
PbBPC3	rna34309	311	35079.63	9.51	58.51	-0.855	nucleus
PbBPC4	rna33161	338	37875.02	9.48	55.69	-0.67	nucleus
PbBPC5	rna38744	339	38137.06	9.34	65.7	-0.992	nucleus
PbBPC6.1	rna51642	340	38085	9.36	59.86	-0.94	nucleus
PbBPC6.2	rna3671	340	38085	9.36	59.86	-0.94	nucleus

### 3.2 Phylogenetic analysis of *BPC* family members

**Figure 1:**
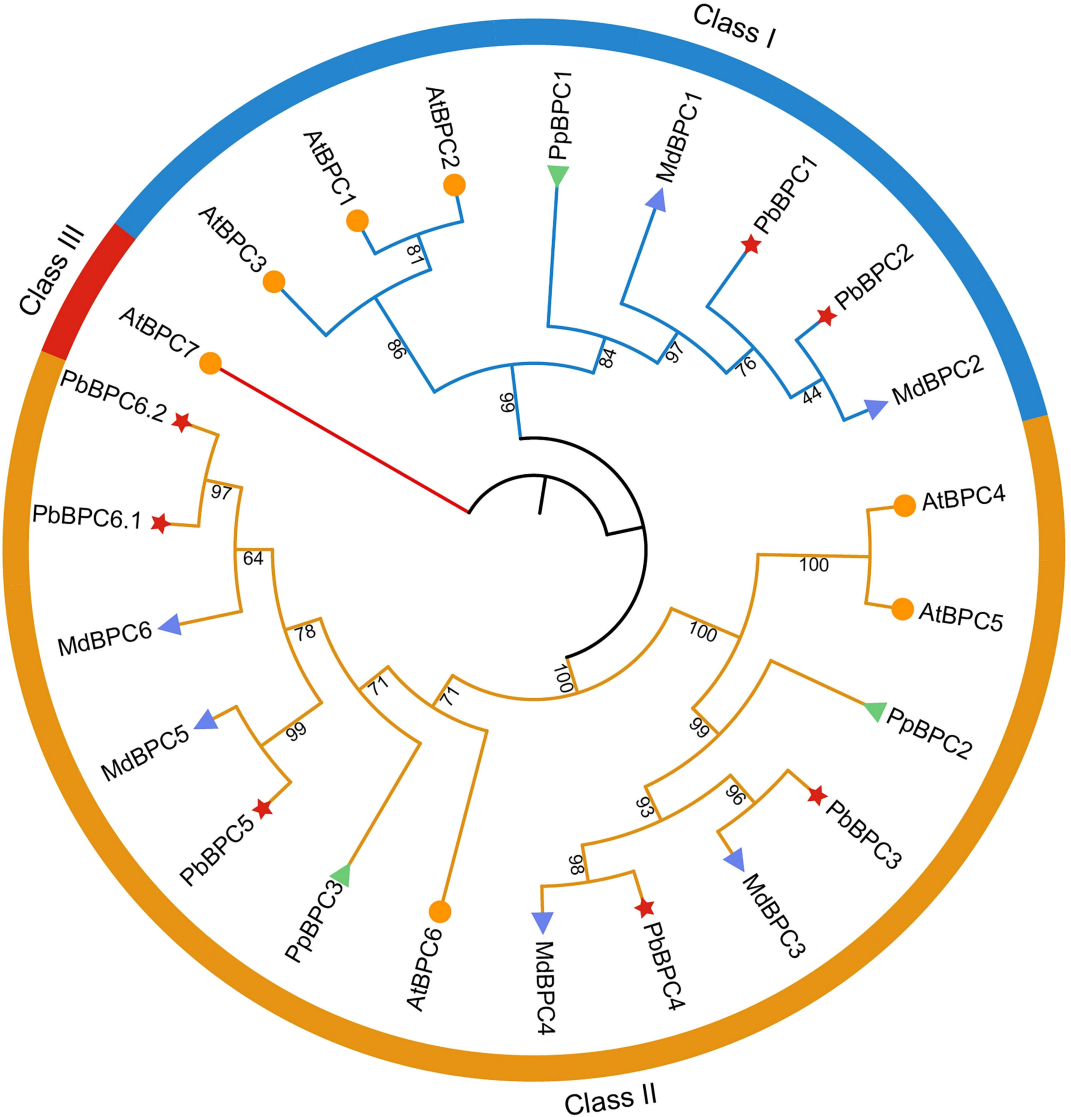
Phylogenetic tree of BPCs in *Pyrus bretschneideri* (Pb), *Arabidopsis thaliana* (At), *Malus domestica* (Md), and *Prunus persica* (Pp).

To investigate the evolutionary relationships of BPC proteins, a phylogenetic tree was constructed using BPC sequences from pear, apple, peach, and Arabidopsis. As shown in **Figure 1**, 23 BPC proteins from the four species were categorized into three distinct classes. Class I comprised eight members, including two pear genes (*PbBPC1* and *PbBPC2*), two apple genes (*MdBPC1* and *MdBPC2*), one peach gene (*PrBPC1*), and three Arabidopsis genes (*AtBPC1*, *AtBPC2*, and *AtBPC3*). Class II contained 14 members, including five pear genes (*PbBPC3*, *PbBPC4*, *PbBPC5*, *PbBPC6.1*, and *PbBPC6.2*), four apple genes (*MdBPC3–6*), two peach genes (*PrBPC2* and *PrBPC3*), and three Arabidopsis genes (*AtBPC4*, *AtBPC5*, and *AtBPC6*). Class III consisted of a single gene, *AtBPC7*, from Arabidopsis. This pattern suggests that Class III BPC genes are more prone to evolutionary loss compared with those in Classes I and II. Within the phylogenetic tree, each pear *BPC* gene clustered most closely with its apple homolog, reflecting their shared ancestry within the Rosaceae family.

### 3.3 Conserved motif and gene structure analysis of *PbBPC* members in pear

**Figure 2:**
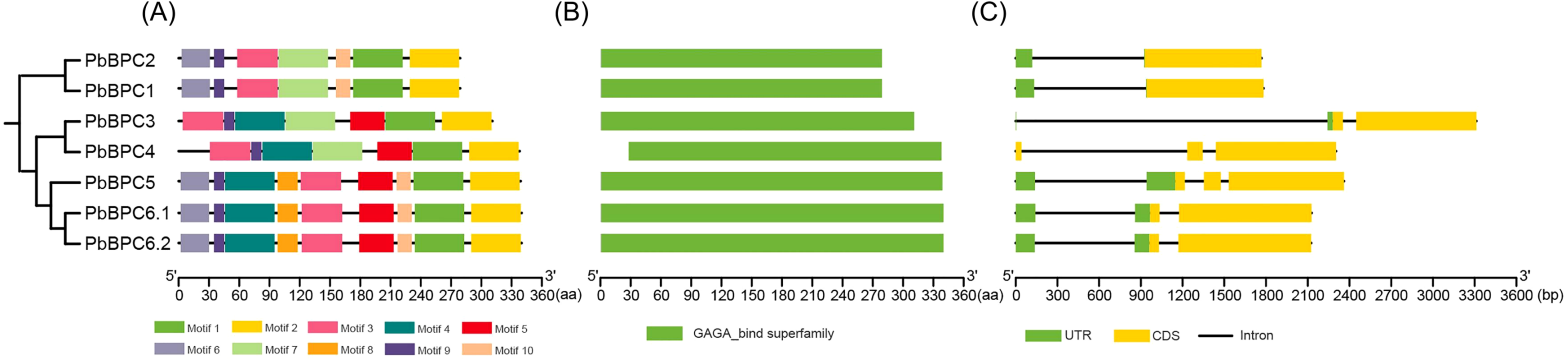
Schematics diagrams of conserved motifs, gene structure, and conserved protein domains of BPC TFs in pear. **(A)** Conserved motifs in PbBPC proteins. **(B)** Conserved domains in PbBPC proteins. **(C)** Gene structures of *PbBPC* members.

To gain deeper insight into *PbBPC* genes, we analyzed their conserved motifs, domains, and gene structures. Motif analysis identified 10 motifs among the seven PbBPC proteins, and their compositions were more similar within each phylogenetic class than between classes (**Figure 2A**). All PbBPC members possessed motifs 1, 2, 3, and 9. Two Class I members (PbBPC1 and PbBPC2) shared an identical composition and order (motifs 6, 9, 3, 7, 10, 1, and 2). In Class II, PbBPC3 and PbBPC4 also had a similar motif order (motifs 3, 9, 4, 7, 5, 1, and 2), whereas PbBPC5, PbBPC6.1, and PbBPC6.2 exhibited highly similar motif compositions and arrangements (**Figure 2A**). All seven proteins contained the GAGA-binding superfamily domain (**Figure 2B**). Gene structure analysis further showed strong conservation within classes. With the exception of *PbBPC4*, all genes possessed untranslated regions (UTRs). *PbBPC1* and *PbBPC2* contained a single exon, whereas most Class II genes had three exons, except *PbBPC3*, which had two (**Figure 2C**). Collectively, these results demonstrate that the phylogenetic classification of *PbBPC* genes is well supported by their motif compositions and gene structures.

### 3.4 Chromosomal locations and synteny analysis of *PbBPC* members in pear

**Figure 3:**
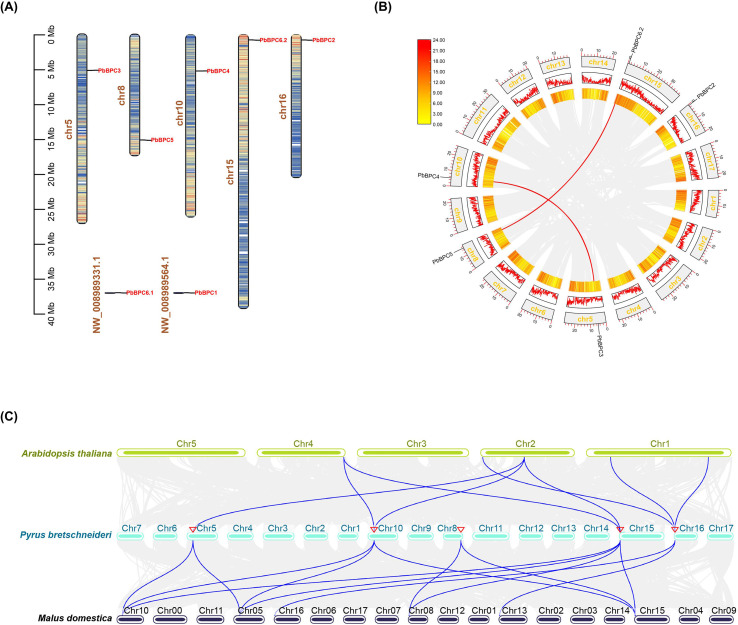
Chromosomal distribution and collinearity analysis of *PbBPC* genes. **(A)** Locations of *PbBPC* genes on five pear chromosomes. **(B)** Intragenomic collinearity of *PbBPC* genes in pear, with duplicated gene pairs connected by red lines. **(C)** Intergenomic collinearity of BPC transcription factors among *Pyrus bretschneideri, Arabidopsis thaliana*, and *Malus domestica*, with collinear gene pairs connected by blue lines.

The chromosomal distribution of *PbBPC* members was examined to clarify their genomic organization. As shown in **Figure 3A**, five *PbBPC* genes were mapped to distinct chromosomes: *PbBPC3* on Chr5, *PbBPC5* on Chr8, *PbBPC4* on Chr10, *PbBPC6.2* on Chr15, and *PbBPC2* on Chr16; *PbBPC1* and *PbBPC6.1* were located on unassembled scaffolds. Collinearity analysis within the pear genome revealed two pairs of segmentally duplicated genes (*PbBPC5/PbBPC6.2* and *PbBPC3/PbBPC4*) (**Figure 3B**). Both duplicated pairs exhibited Ka/Ks ratios less than 1, indicating that they mainly underwent purifying selection (**Supplementary Table S2**). To further explore evolutionary relationships across species, collinearity analysis was conducted among *P. bretschneideri*, *A. thaliana*, and *M. domestica*. *PbBPC2*, *PbBPC3*, *PbBPC4*, and *PbBPC6.2* showed strong collinearity with BPC genes in Arabidopsis, while 13 collinear gene pairs were identified between pear and apple, reflecting high homology and strong conservation within the Rosaceae family (**Figure 3C**).

### 3.5 Analysis of *cis*-elements in the upstream sequences of *BPC* genes in pear

**Figure 4:**
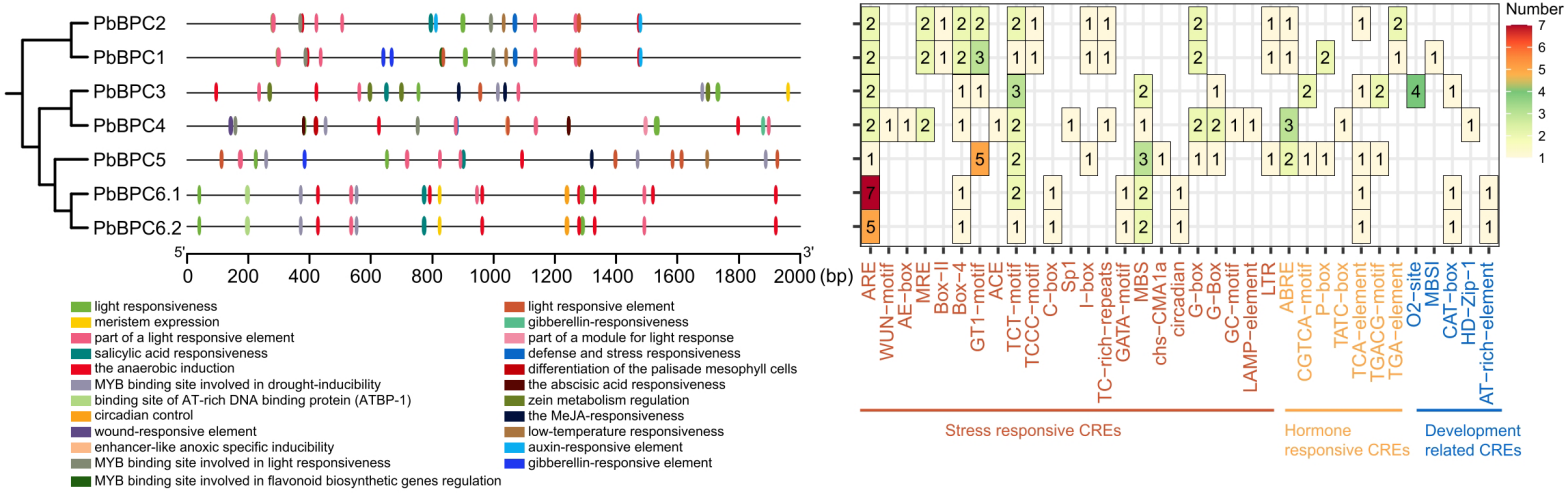
Analysis of cis-acting elements in the promoter regions of *PbBPC* genes.

To better understand the potential functions and regulatory mechanisms of the *PbBPC* gene family, *cis*-acting elements were analyzed in the 2,000-bp promoter regions upstream of each *PbBPC* gene. This analysis identified the presence of development-related *cis*-elements, including the CAT-box, AT-rich element, and O2-site, underscoring the potential involvement of *PbBPC* genes in the regulation of plant development (**Figure 4**). Furthermore, the promoters were found to contain abundant stress-responsive elements, such as ARE (anaerobic induction), MBS (MYB-binding site involved in drought inducibility), and TC-rich elements (defense and stress response), among others. Additionally, several hormone-responsive elements with known links to stress regulation were identified, including ABRE (abscisic acid responsiveness), TCA-element (salicylic acid responsiveness), and TGACG-motif (MeJA responsiveness) (**Figure 4**). The presence of drought-related *cis*-elements indicates that specific *PbBPC* genes may play important roles in modulating drought tolerance in pear.

### 3.6 Expression of *BPC* genes in different tissues of pear and under dehydration treatment

**Figure 5:**
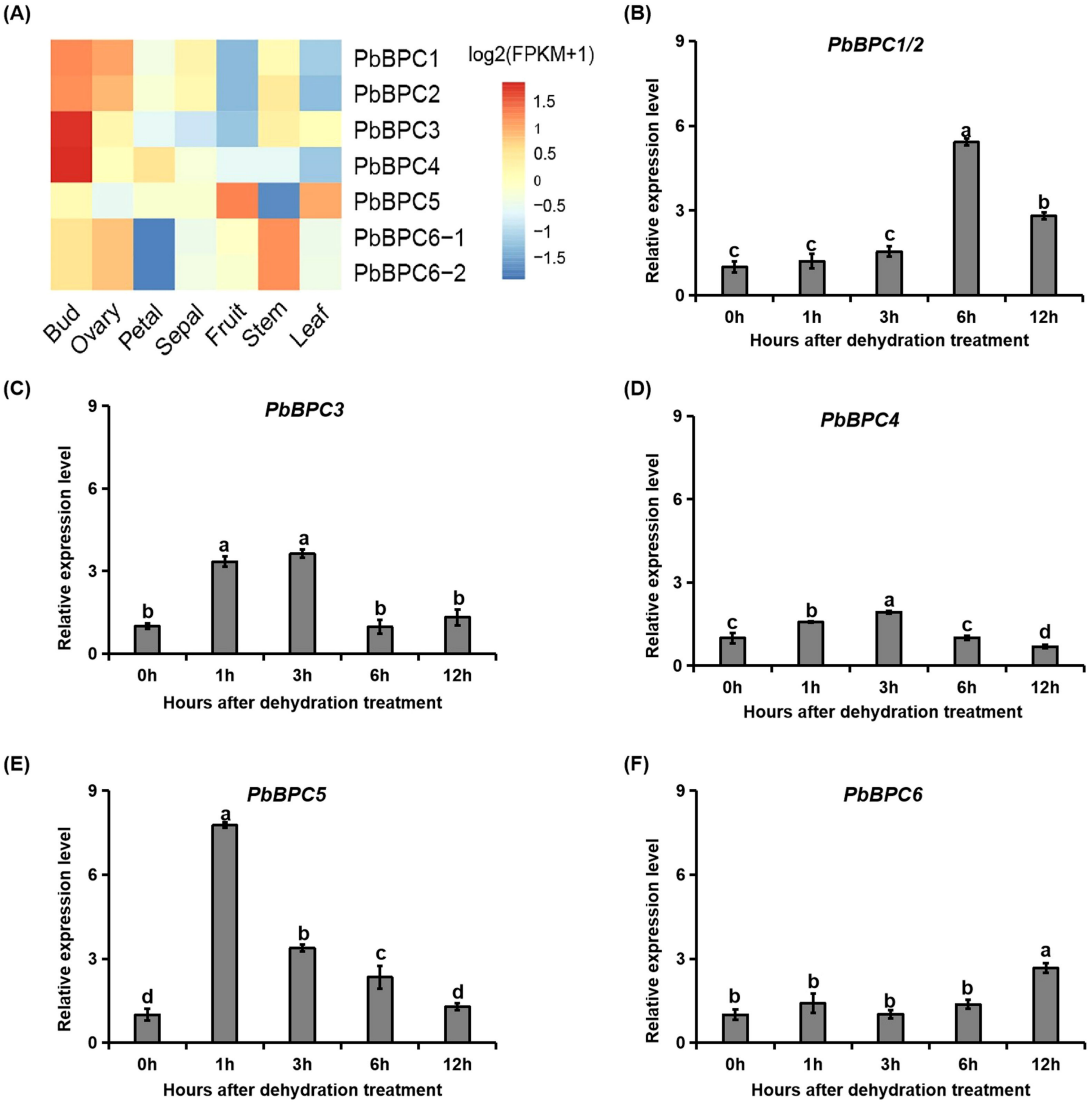
Expression patterns of *PbBPC* genes in pear. **(A)** Heatmap of RNA-seq expression of *PbBPC* genes across different pear tissues. **(B-F)** Expression patterns of *PbBPC* genes in leaves under dehydration treatment. Data represent mean ± SD (n=3). Different letters denote statistically significant differences (*P* < 0.05, Tukey’s multiple range test).

To investigate the expression patterns of *PbBPC* genes across pear tissues, transcriptome data from the bud, fruit, leaf, ovary, petal, sepal, and stem of Chinese white pear were analyzed. Most *PbBPC* genes showed high expression in bud and ovary, particularly *PbBPC1*, *PbBPC2*, *PbBPC3*, and *PbBPC4*, suggesting that they play a role in early growth and reproductive development (**Figure 5A**). *PbBPC5* exhibited relatively high expression in leaf and fruit, whereas other members displayed low expression in these tissues. Notably, *PbBPC6.1/6.2* were more highly expressed in the stem than in other tissues (**Figure 5A**). Because promoter analysis revealed that most *PbBPC* genes contained *cis*-elements associated with the drought response, their expression under dehydration treatment was further examined. Under dehydration treatment, the transcript levels of *PbBPC* genes were generally increased. *PbBPC1/2*, *PbBPC3*, *PbBPC4*, and *PbBPC5* showed an initial rise followed by a decline, while *PbBPC6.1/6.2* steadily increased, reaching 2.6-fold higher expression at 12 h compared with 0 h. Among them, *PbBPC5* responded most rapidly and strongly, peaking at 1 h after dehydration with a 7.8-fold increase relative to the expression level at 0 h (**Figures 5B-F**). *PbBPC5* was thus selected for functional studies of the role of BPC transcription factors in drought tolerance.

### 3.7 Subcellular localization of PbBPC5

**Figure 6:**
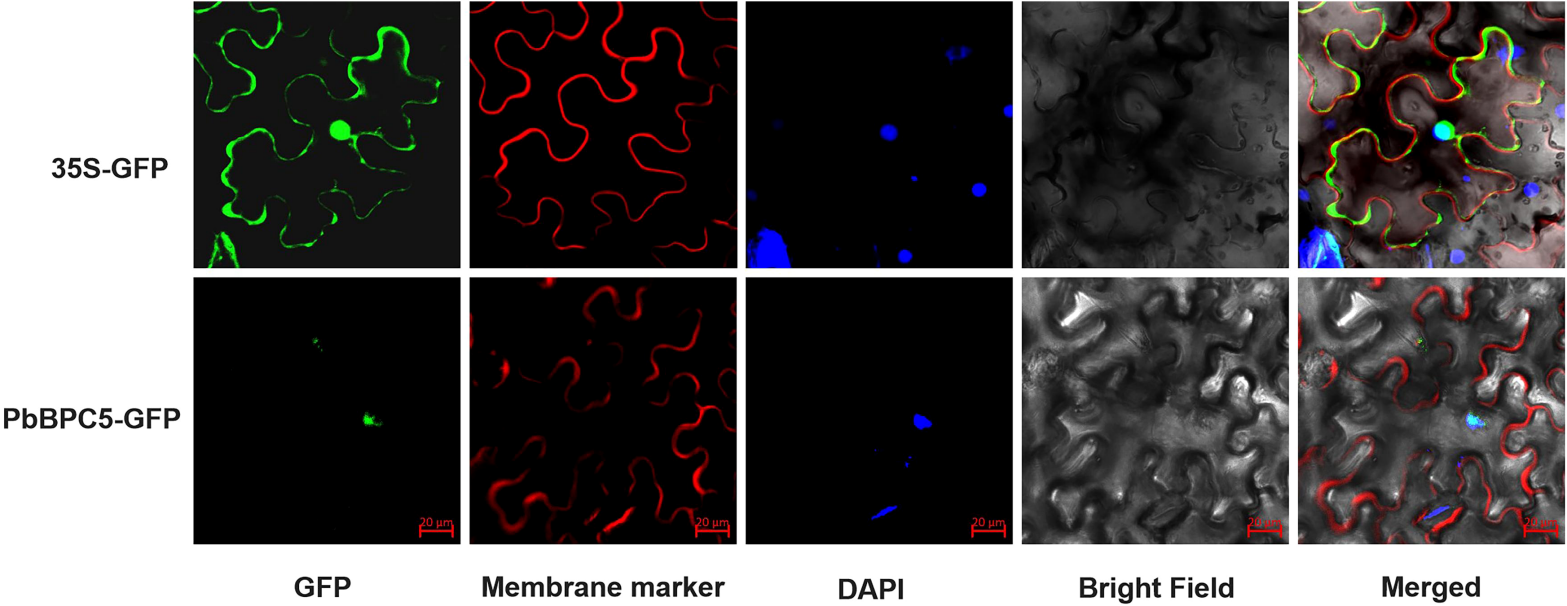
Subcellular localization of PbBPC5.

As a transcription factor, *PbBPC5* was predicted to function primarily within the nucleus. To verify its subcellular localization, the PbBPC5::GFP fusion construct was co-expressed with the membrane marker CBL1n::OFP in *Nicotiana benthamiana* leaf cells. Previous studies have shown that CBL1n::OFP is localized to the plasma membrane and was therefore used as a membrane marker in this experiment (Batistic et al., 2010). As shown in **Figure 6**, fluorescence from PbBPC5::GFP was detected in the nucleus, and DAPI staining further confirmed the nuclear localization. These results demonstrate that PbBPC5 is indeed a nuclear protein, consistent with its predicted role as a transcription factor.

### 3.8 Silencing of *PbBPC5* reduced the drought tolerance of pear

**Figure 7:**
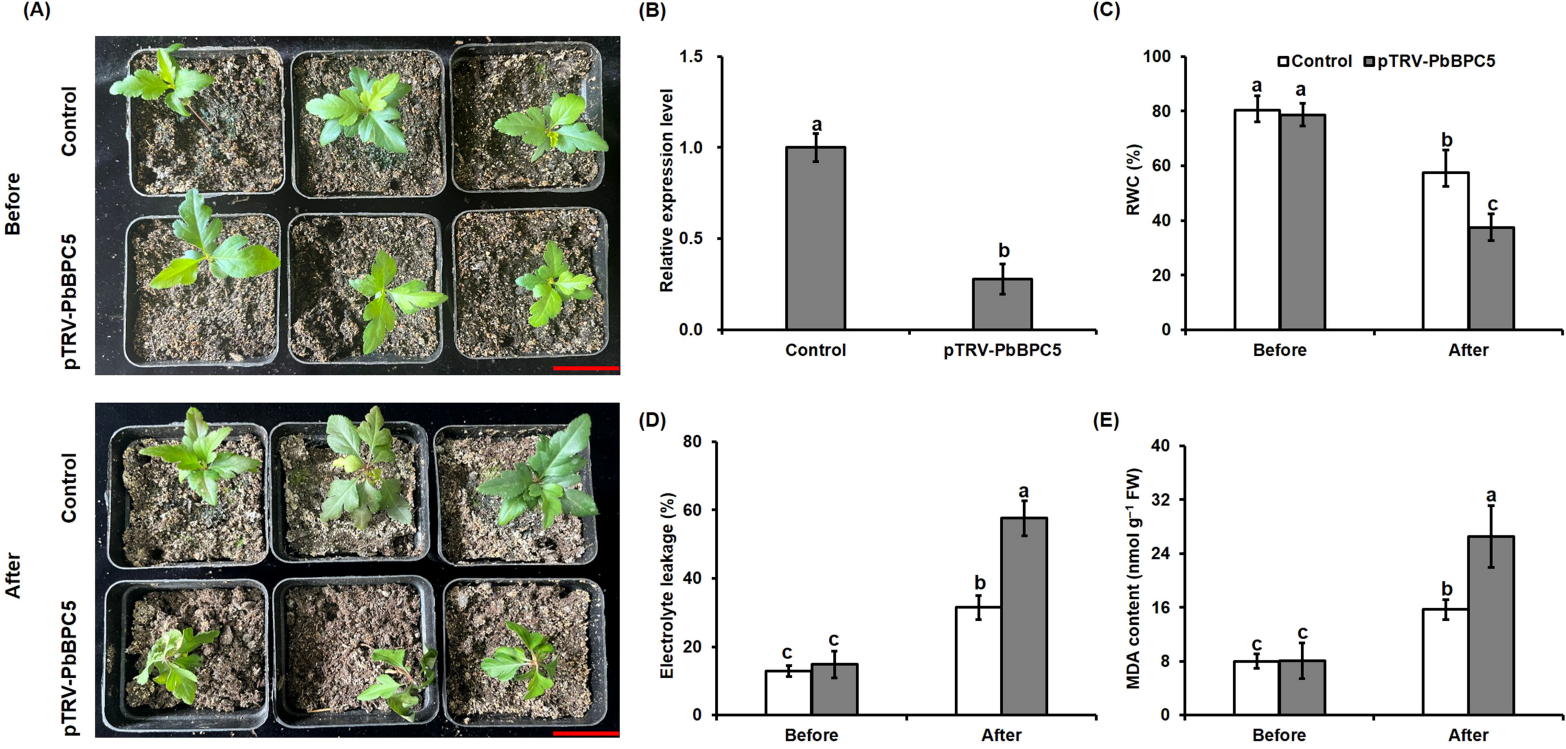
Effect of PbBPC5 silencing on the drought tolerance of pear. **(A)** Comparative phenotypic analysis of control and PbBPC5-silenced pear plants prior to and following a 20-day drought treatment. **(B)** The expression level of PbBPC5 was measured in both control and PbBPC5-silenced pear plants after 7 days of drought exposure. **(C)** RWC, **(D)** relative electrolyte leakage, and **(E)** MDA content in leaves of control and PbBPC5-silenced pear plants before and after drought treatment. Scale bar: 3cm. Data represent mean ± SD (n = 3). Different letters indicate statistically significant differences (P < 0.05, Tukey’s multiple range test).

To elucidate the function of *PbBPC5* in regulating drought tolerance in pear, a VIGS assay was performed to transiently silence *PbBPC5* in *P. betulaefolia*. *PbBPC5* transcript levels were approximately 70% lower in pTRV*-PbBPC5* plants than in control plants (**Figure 7B**). Under normal conditions, no phenotypic differences in growth were observed between the control and pTRV*-PbBPC5* plants. However, after 20 days of drought stress, pTRV*-PbBPC5* plants displayed more pronounced wilting and leaf curling than controls (**Figure 7A**). Physiological indices commonly used to assess drought damage, including RWC, electrolyte leakage, and malondialdehyde (MDA) content, were measured. Prior to drought treatment, no significant differences were detected between groups. Following drought, RWC was significantly reduced in pTRV-*PbBPC5* plants relative to control plants, while both electrolyte leakage and the MDA content increased markedly in all plants, with greater increases in silenced lines compared with controls (**Figures 7C-E**). These results suggest that silencing *PbBPC5* reduced drought tolerance in pear.

### 3.9 *PbBPC5* affects ROS metabolism in pear under drought stress

**Figure 8:**
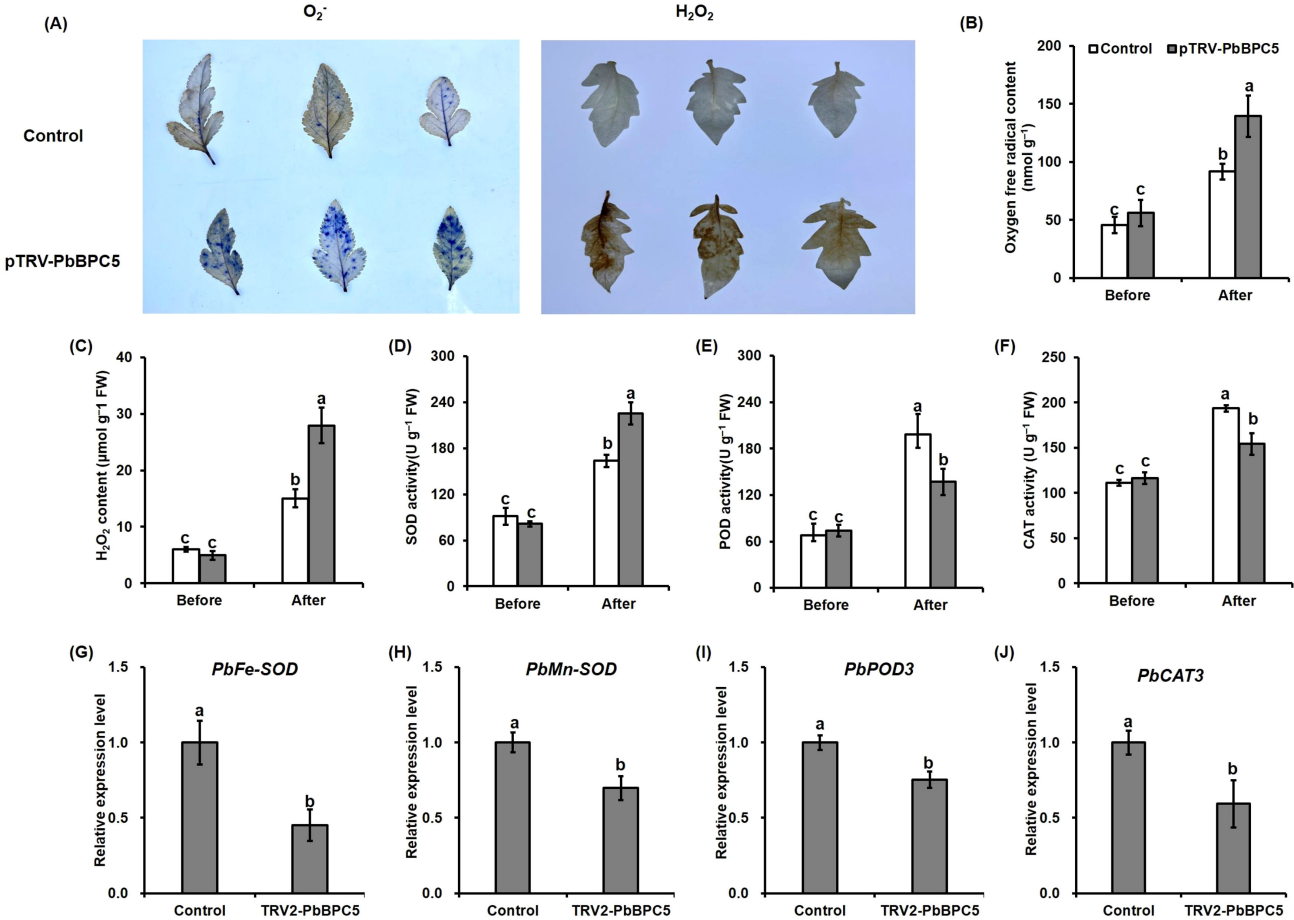
Effect of PbBPC5 silencing on the antioxidant defense of pear under drought stress. **(A)** Visualization of O_2_^−^ and H_2_O_2_ accumulation in the leaves of control and PbBPC5-silenced pear plants before and after drought treatment by NBT and DAB staining, respectively. **(B)** O_2_^–^ content, **(C)** H_2_O_2_ content, **(D)** SOD activity, **(E)** POD activity, and **(F)** CAT activity in leaves of control and PbBPC5-silenced plants before and after drought treatment. Expression levels of **(G)** PbFe-SOD, **(H)** PbMn-SOD, **(I)** PbPOD3, and **(J)** PbCAT3 in leaves of control and PbBPC5-silenced plants after drought treatment. Data represent mean ± SD (n = 3). Different letters indicate statistically significant differences (P < 0.05, Tukey’s multiple range test).

Drought is known to cause excessive ROS accumulation, resulting in oxidative damage to plant cells. After 20 days of drought treatment, leaves from both control and pTRV*-PbBPC5* plants were subjected to NBT and DAB staining to visualize O_2_^–^ and H_2_O_2_ accumulation, respectively. As shown in **Figure 8A**, pTRV*-PbBPC5* plants exhibited more intense blue staining with NBT and stronger brown coloration with DAB compared with control plants under drought stress, indicating higher ROS levels (**Figure 8A**). Consistent with these results, quantitative assays revealed that O_2_^−^ and H_2_O_2_ concentrations were significantly elevated in silenced plants compared with control plants under drought stress, whereas no differences were observed before treatment (**Figures 8B, C**). To assess antioxidant capacity, the activities of SOD, POD, and CAT were measured. Prior to drought, enzyme activities were comparable between groups; however, under stress, all three enzyme activities were markedly lower in pTRV*-PbBPC5* plants (**Figures 8D–F**). In addition, the expression levels of genes related to antioxidant enzymes, including *PbFe-SOD*, *PbMn-SOD*, *PbPOD3*, and *PbCAT3*, were significantly reduced in silenced lines relative to controls under drought stress (**Figures 8G–J**). Together, these results suggest that silencing *PbBPC5* decreases antioxidant capacity in pear, thereby reducing drought tolerance.

## 4 Discussion

In recent years, the combined effects of global warming and groundwater scarcity have intensified the adverse effects of drought on agricultural productivity (Jury and Vaux, 2005). Transcription factors are widely recognized as key regulators of plant responses to multiple environmental stresses (Xiao et al., 2017). BPC proteins comprise a plant-specific transcription factor family implicated in the regulation of plant growth and development, as well as in responses to unfavorable environments (Mu et al., 2017a, Mu et al., 2017b; Theune et al., 2017; Sahu et al., 2023). BPC family members have been identified in multiple plant species, and the number of *BPC* genes varies considerably among species. For example, 4, 7, 12, 25, and 8 BPC members have been reported in Arabidopsis (Meister et al., 2004), *Cucumis sativus* (Li et al., 2019), *Brassica campestris* (Zhang et al., 2023), *Brassica napus* (Wang et al., 2024), and *Cocos nucifera* (Lao et al., 2024), respectively. However, studies examining BPC family members in pear and their roles in stress responses remain limited. We systematically characterized *PbBPC* genes in the pear genome, examined their expression in various tissues and under dehydration, and further validated the role of *PbBPC5* in drought tolerance using VIGS technology. Our results expand our knowledge of the BPC family in pear and provide valuable genetic resources for drought-resistance breeding.

Seven *PbBPC* members were identified in *P. bretschneideri* and compared with homologs from apple, peach, and Arabidopsis. Phylogenetic analysis grouped these 23 proteins into three classes, consistent with the established classification in Arabidopsis (Meister et al., 2004). All PbBPC proteins were assigned to Class I and Class II, consistent with their classification in apple and peach. Similar patterns have been reported in cucumber, where four BPC proteins were divided into two groups (Li et al., 2019). This suggests that Class III members may have been lost during evolution. Supporting this classification, analyses of gene structures and conserved motifs revealed strong similarities in gene structure and motif organization among *PbBPC* members within the same subgroup, indicating close evolutionary relationships among members within the same subgroup. Comparable findings in other species indicate that BPC genes are highly conserved within subgroups but divergent across groups (Zhu et al., 2025; Pang et al., 2025). Previous studies suggest that the variable N-terminal domains of BPC proteins form coiled-coil structures involved in dimerization, protein–protein interactions, and nucleolar localization (Kooiker et al., 2005; Gong et al., 2018). In our analysis, N-terminal motifs varied between subgroups, while motifs 1 and 2 at the C-terminus were highly conserved across all *PbBPC* proteins. These motifs contained five conserved cysteine residues predicted to form a zinc finger-like structure for recognizing GAGA motifs, although alternative mechanisms have also been proposed (Monfared et al., 2011; Theune et al., 2017; Ma et al., 2022).

Gene family expansion in plants is primarily driven by tandem and segmental duplications (Cannon et al., 2004; Song et al., 2016). To evaluate the contribution of these duplication events to *PbBPC* gene expansion, we conducted a collinearity analysis of the *PbBPC* gene family. Two segmental duplications involving *PbBPC* genes were identified, suggesting that the family did not expand extensively during pear evolution. Similarly, limited expansions were observed in grape, coconut, cucumber, and camellia (Li et al., 2019; Zhang et al., 2025; Lao et al., 2024; Yu et al., 2025). Comparative collinearity analysis showed stronger homology of *PbBPC* genes with apple than with Arabidopsis, reflecting their closer evolutionary relationship within the Rosaceae family and suggesting potentially conserved functions among related crops.

Gene expression patterns are typically correlated with the types and numbers of *cis*-regulatory elements present in promoter regions (Eerapagula et al., 2025). Previous studies have shown that BPC TFs regulate diverse plant growth and development processes (Sahu et al., 2023). Mutations in BPC genes can cause diverse vegetative and reproductive abnormalities, such as dwarfism, small leaves, and abnormal ovules (Monfared et al., 2011; Simonini and Kater, 2014). BPC1 and BPC2 affect ovule development and the growth of endosperm and embryos in Arabidopsis by suppressing *FUSCA3* expression (Wu et al., 2020). Consistent with these findings, the promoter regions of *PbBPC* members comprised several *cis*-regulatory elements linked to plant growth and development, including CAT-box, AT-rich element, and O2-site (Wang et al., 2024). Studies of tissue-specific expression provide valuable insights into gene functions during plant growth and development (Li et al., 2019). We found that most *PbBPC* members were highly expressed in buds and ovaries, indicating that they might play a role in the early growth and reproductive stages of pear.

In addition, the promoter regions of *PbBPC* contained numerous stress-related *cis*-acting elements, especially in *PbBPC5*, which harbored multiple ABRE and MBS motifs. Previous studies showed contrasting roles of BPC proteins in stress responses; for example, *CsBPC2* enhanced salt tolerance in cucumber by regulating ROS metabolism, osmotic adjustment, and ion balance (Li et al., 2023), whereas *AtBPC2* suppressed osmotic stress resistance in Arabidopsis by downregulating *LEA4-5* (Li et al., 2021). Although numerous studies have revealed that BPC TFs respond to diverse environmental stresses across multiple plant species, their roles in regulating stress tolerance and the underlying mechanisms remain poorly understood (Hu et al., 2020; Xian et al., 2020; Zhang et al., 2023; Sahu et al., 2023; Wang et al., 2024). We analyzed the transcriptional responses of *PbBPC* genes during dehydration. All *PbBPC* genes were induced to some degree, with *PbBPC5* displaying the most pronounced response. Given the enrichment of drought-responsive elements in its promoter and its strong induction during dehydration, *PbBPC5* was selected for functional analysis to further clarify its role in drought resistance in pear.

Drought stress disrupts multiple physiological and biochemical processes and is a major cause of yield reduction in crops (Zhu, 2016). To explore the role of *PbBPC5* in regulating the drought response, both control plants and *PbBPC5*-silenced plants were subjected to drought stress. Silencing *PbBPC5* significantly weakened pear drought tolerance, as evidenced by reduced RWC, increased electrolyte leakage, and elevated MDA levels compared with control plants. These indicators reflect compromised cell membrane stability, a hallmark of stress-induced cellular injury (Bajji et al., 2004; Jia et al., 2021a). These results demonstrated that *PbBPC5* silencing in pear could exacerbate the cellular damage inflicted by drought stress. ROS overaccumulation is another key factor contributing to drought-induced damage (Wang et al., 2016), and enhanced ROS metabolism has been strongly associated with improved drought tolerance in many plants (Sun et al., 2018; Wang et al., 2019; Zhao et al., 2020; Huang et al., 2021; Jia et al., 2021a, Jia et al., 2021b). For example, *MdATG8i* enhanced apple drought tolerance by promoting ROS scavenging (Jia et al., 2021a). In our study, *PbBPC5* silencing increased ROS accumulation, reduced antioxidant enzyme activities (SOD, POD, CAT), and downregulated antioxidant enzyme-related enzymes (*PbFe-SOD*, *PbMn-SOD*, *PbPOD3*, *PbCAT3*), indicating that compromised antioxidant defenses in pTRV-*PbBPC5* plants led to greater cellular damage, increasing drought susceptibility. This is consistent with recent research suggesting that BPC TFs regulate ROS homeostasis under stress, such as *CsBPC2*, which positively regulates salt stress resistance in cucumber by modulating ROS scavenging (Li et al., 2023), and *BcBPC9*, which promotes the expression of antioxidant enzyme genes under Cd stress (Zhang et al., 2023). Together, these findings demonstrate that *PbBPC5* silencing reduces drought resistance in pear by weakening ROS scavenging and disrupting cellular homeostasis.

## 5 Conclusions

In this study, we conducted a systematic analysis of the BPC gene family in pear, identifying seven *PbBPC* members. Using comprehensive bioinformatics approaches, we examined their phylogenetic relationships, conserved motifs, gene structures, protein domains, chromosomal distributions, collinearity, and promoter *cis*-elements. Given the enrichment of drought-responsive *cis*-elements in their promoters, we further investigated their expression under dehydration treatment. Most *PbBPC* genes were upregulated, with *PbBPC5* showing the strongest induction. Functional validation using VIGS revealed that silencing *PbBPC5* reduced drought tolerance, as pTRV*-PbBPC5* plants accumulated more ROS and experienced greater cellular damage than controls. Together, these findings offer novel insights into the evolutionary and functional characteristics of the *PbBPC* gene family and identify *PbBPC5* as a potential positive regulator of drought tolerance in pear. This work will aid future research on the roles of BPC transcription factors in stress adaptation and provide valuable genetic resources for enhancing drought resistance in pear and related species.

## Data Availability

The datasets presented in this study can be found in online repositories. The names of the repository/repositories and accession number(s) can be found in the article/**Supplementary Material**.
