# Supplementary material for: Genome-wide characterization of BPC transcription factors in pear and functional validation of PbBPC5 in drought tolerance regulation
Source: Front Plant Sci. 2026 Jan 21;16:1752990. doi: 10.3389/fpls.2025.1752990 (PMC12868192; doi:10.3389/fpls.2025.1752990)
Supplement: Supplementary file 1 [file Table1.docx]

Supplementary Material

**Table S1** Primers used in this study

| Primer name | Sequence |
| --- | --- |
| PbBPC5-F | ATGGATGACGGTGGCCATCG |
| PbBPC5-R | CTACTTGATTGTGATGTAGCGG |
| pTRV2-PbBPC5-F | TGAGTAAGGTTACCGAATTC AGCTATTCAAGAAAGAAA |
| pTRV2-PbBPC5-R | GTGAGCTCGGTACCGGATCC TATCCCTCGTACCATAAG |
| PbBPC5-GFP-F | CATATGCCCGTCGACATGGATGACGGTGGCCATCGTG |
| PbBPC5-GFP-R | GCCCTTGCTCACCATCTTGATTGTGATGTAGCGGTT |
| qPbBPC1/2-F | TCAGTTCCTCGTGTGAAGCC |
| qPbBPC1/2-R | CTGTTGTGCAACTCCAGTGC |
| qPbBPC3-F | CAGTGCCAGTGTGTTCATGC |
| qPbBPC3-R | GACATATGGGTGGGGCAACA |
| qPbBPC4-F | CGCCCCTCAATCCATTCAGT |
| qPbBPC4-R | TTTGCACGCTTTGTCTGACG |
| qPbBPC5-F | TCAGGGTCAGTGGTTGATGC |
| qPbBPC5-R | TCTCGTTGCAGGAATGCCAT |
| qPbBPC6.1/6.2-F | AGGATATGGGCAGTGGAGGT |
| qPbBPC6.1/6.2-R | CCCCAGGTCCTGACATTTCC |
| qPbFe-SOD-F | CTTCGAATGCCATTTGCCCA |
| qPbFe-SOD-R | GCCGCATTCCAAGACACAAG |
| qPbMn-SOD-F | ATCAGGGTTCCAGAGCAAGC |
| qPbMn-SOD-R | CAGAGCGCCATAGTCGTAGG |
| qPbPOD3-F | GATATGCCCGCAGAATGGGA |
| qPbPOD3-R | AGCCCCGCTAGTTGAAAACA |
| qPbCAT3-F | ACGTGCTGACTTCCTTCGAG |
| qPbCAT3-R | AGTTTCAAGGCTGCCACACT |
| Tubulin-F | TGGGCTTTGCTCCTCTTAC |
| Tubulin-R | CCTTCGTGCTCATCTTACC |

**Table S2** Ka/Ks analysis for *BPC* duplicated genes of pear.

| **Sequence** | **Ka** | **Ks** | **Ka/Ks** |
| --- | --- | --- | --- |
| PbBPC3&PbBPC3 | 0.038893848 | 0.113800814 | 0.341771262 |
| PbBPC5&PbBPC6.2 | 0.02546934 | 0.216143331 | 0.117835421 |
